# Risk for Infection in Humans after Exposure to Birds Infected with Highly Pathogenic Avian Influenza A(H5N1) Virus, United States, 2022

**DOI:** 10.3201/eid2906.230103

**Published:** 2023-06

**Authors:** Krista Kniss, Kelsey M. Sumner, Katie J. Tastad, Nathaniel M. Lewis, Lauren Jansen, Derek Julian, Mike Reh, Emily Carlson, Robin Williams, Samir Koirala, Bryan Buss, Matthew Donahue, Jennifer Palm, Leslie Kollmann, Stacy Holzbauer, Min Z. Levine, Todd Davis, John R. Barnes, Brendan Flannery, Lynnette Brammer, Alicia Fry

**Affiliations:** Centers for Disease Control and Prevention, Atlanta, Georgia, USA (K. Kniss, K.M. Sumner, K.J. Tastad, N.M. Lewis, L. Jansen, M. Reh, B. Buss, S. Holzbauer, M.Z. Levine, T. Davis, J.R. Barnes, B. Flannery, L. Brammer, A. Fry);; Nebraska Department of Health and Human Services, Lincoln, Nebraska, USA (L. Jansen, D. Julian, M. Reh, E. Carlson, R. Williams, S. Koirala, B. Buss, M. Donahue);; Minnesota Department of Health, Saint Paul, Minnesota, USA (J. Palm, L. Kollmann, S. Holzbauer)

**Keywords:** influenza, highly pathogenic avian influenza, epidemiology, farms, surveillance systems, viruses, respiratory infections, zoonoses, bird flu, United States

## Abstract

During February 7─September 3, 2022, a total of 39 US states experienced outbreaks of highly pathogenic avian influenza A(H5N1) virus in birds from commercial poultry farms and backyard flocks. Among persons exposed to infected birds, highly pathogenic avian influenza A(H5) viral RNA was detected in 1 respiratory specimen from 1 person.

Infection with highly pathogenic avian influenza (HPAI) virus results in high mortality rates in chickens, gallinaceous birds, and some wild bird species (*1*). Potential for HPAI virus transmission and adaptation to human hosts poses a pandemic risk (*2*). In 2021, HPAI viruses belonging to influenza A(H5N1) clade 2.3.4.4b were detected worldwide in migrating birds and commercial poultry flocks (*3*). HPAI H5N1 viruses were first detected in the United States in January 2022 in hunter-harvested wild birds in North and South Carolina (*4*); reports of infected wild and domesticated birds in other states followed. Infection risk among persons exposed to birds with H5N1 clade 2.3.4.4b infection is unknown, although 2 human cases were reported in China, 1 in Chile, 1 in Ecuador, 2 in Spain, and 1 in the United Kingdom (*5*,*6*). Using active symptom monitoring of exposed persons in the United States during February 7–September 3, 2022, we estimated the risk for symptomatic H5N1 virus infection in humans and developed a surveillance protocol for monitoring asymptomatic infection by using serologic testing among persons exposed to H5N1-infected birds.

## The Study

In the United States, the US Department of Agriculture is responsible for conducting surveillance for avian influenza in wild or domesticated birds (*7*). An outbreak of HPAI in domesticated or commercial flocks was defined as >1 case of laboratory-confirmed avian influenza in a bird. For persons exposed (e.g., flock owners, farm workers, and cullers) to commercial poultry, backyard flocks, wild birds, and the environments of birds infected with HPAI, the Centers for Disease Control and Prevention (CDC) recommended active symptom monitoring (conducted through a mixture of phone, email, and text contact based on the jurisdiction’s preference) by health departments for 10 days after their most recent exposure among persons who did not wear recommended personal protective equipment (PPE) or had a breach in PPE (*8*). State and local health departments used different criteria to determine whether a person met the criteria for active monitoring, and PPE use data may have been collected by state and local health departments but was not collected by CDC. Respiratory specimens (typically nasal or nasopharyngeal swabs) were collected from persons with symptoms compatible with influenza A(H5) virus infection within 10 days of their most recent exposure and tested for influenza A(H5) by real-time reverse transcription PCR (rRT-PCR) at state public health labs using the CDC Human Influenza Virus Real-Time RT-PCR Diagnostic Panel, Influenza A(H5) Subtyping Kit (*9*). Jurisdictions could also test persons without compatible symptoms at their discretion. Any influenza A(H5)–positive results from states were confirmed by testing at CDC. Confirmed diagnostic positive samples were characterized at CDC by using genomic sequencing and viral culture to determine if samples contained infectious influenza A(H5) virus. CDC collected aggregate data from state health departments, including the number of persons monitored and tested for influenza A(H5).

To assess the risk for asymptomatic human infection, alongside state and local health departments in Nebraska and Minnesota, CDC collected serum and respiratory specimens to detect influenza A(H5) virus infection among asymptomatic and symptomatic persons exposed to H5N1-infected poultry in commercial farms, backyard flocks, and wildlife rehabilitation centers experiencing animal outbreaks. All exposed persons were invited to participate in collection of acute respiratory specimens for rRT-PCR diagnostic testing of influenza A(H5) and paired acute and convalescent serum specimens collected 3–4 weeks apart for hemagglutination inhibition and microneutralization assays against A/American Wigeon/South Carolina/22-000345-001/2021 2.3.4.4.b A(H5N1) virus (*10*). Activities were conducted as part of a public health response and not considered human subjects research under federal human subject protection regulations.

During February 7–September 3, 2022, HPAI H5N1 virus infections were detected in 2,199 wild birds in 45 US states. The US Department of Agriculture’s National Veterinary Services Laboratory also confirmed H5N1 outbreaks in 200 commercial poultry farms and 229 backyard flocks in 39 states (Figure). Nationally, 4,351 persons were actively monitored after exposure to these birds, and 3,658 (84%) completed the 10-day monitoring period (Table). Among persons monitored for postexposure symptoms, 134 (3%) experienced onset of >1 symptoms compatible with influenza virus infection and had respiratory specimens collected for diagnostic testing. All 134 symptomatic persons reported mild illness. One person in Colorado with reported fatigue tested positive for A(H5) by rRT-PCR (*11*).

**Figure:**
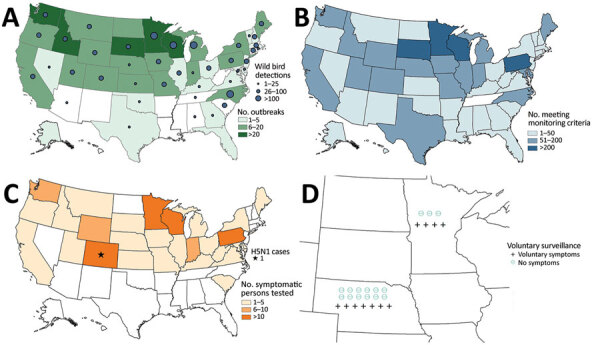
Highly pathogenic avian influenza A(H5N1) virus infection outbreaks and human exposures, United States, February 7–September 3, 2022. A) Number of outbreaks in commercial poultry and backyard flocks and number of detections of H5N1 among wild birds. B) Number of persons exposed and meeting active monitoring criteria. C) Number of persons who were symptomatic during 10-day monitoring period and number of influenza A(H5N1) virus infection cases reported. D) Number of persons in Nebraska and Minnesota who expressed interest in asymptomatic and symptomatic serologic surveillance.

**Table:** Characteristics of exposed persons monitored and tested for influenza A(H5) virus after exposure to highly pathogenic avian influenza A(H5N1) virus–infected birds, United States, February 7–September 3, 2022*

Characteristic	Met jurisdiction-level active monitoring criteria	Completed 10-day monitoring period	Tested through surveillance	Expressed interest in asymptomatic and symptomatic serologic surveillance
Total	4,351 (100)	3,658 (100)	154 (100)	26 (100)
Demographic characteristics				
Median age (range), y			40 (0.5–79)	40.5 (9–73)
Sex				
M				13 (50)
F				13 (50)
Symptomatic†			134 (93)	11 (42)
Hospitalized			0	0
Exposure category				
Farm worker or owner and other nonresponders	1,219 (28)	1,114 (30)	36 (27)‡	
Responders§	2,072 (48)	1,839 (50)	76 (57)‡	
Other, e.g., wildlife, veterinarian, laboratorian	87 (2)	86 (2)	22 (16)‡	
Unknown	973 (22)	619 (17)	0‡	

*Values are no. (%) except as indicated. †Having >1 symptoms compatible with A(H5) infection: fever or feeling feverish or chills; cough; sore throat; runny or stuffy nose; eye tearing, redness, irritation (pink eye); sneezing; difficulty breathing; shortness of breath; fatigue (feeling very tired); muscle or body aches; headaches; nausea; vomiting; diarrhea; seizures; or rash. Ten exposed but asymptomatic persons and 10 persons without symptom history available had respiratory specimens collected under their jurisdiction’s discretion or in conjunction with follow-up to the single human case detected. ‡Exposure category information only collected through surveillance for persons who reported being symptomatic. §Defined as persons responsible for performing response activities such as culling, cleaning, and decontaminating infected premises.

Twenty-six persons with exposure to H5N1-infected birds in 5 investigations in Nebraska and 1 investigation in Minnesota expressed interest in the additional serum and respiratory swab sample collection, including 11 (42%) persons who reported symptoms after contact with sick birds. Nasal swab samples and paired serum specimens were obtained from 17 persons, and nasal swab samples only were obtained from 5 persons; 4 persons had no specimens tested. All 22 persons with collected nasal swab samples tested negative for influenza viruses by rRT-PCR. The 17 persons with paired serum specimens demonstrated no increase in antibody titers to influenza A(H5) 2.3.4.4b virus. Nineteen participants were present for culling of sick birds, and all reported PPE use of variable type and duration.

## Conclusions

More than 4,000 persons exposed to HPAI H5N1–infected birds were monitored for symptomatic illness across the United States, and only 1 rRT-PCR–confirmed influenza A(H5) case was detected in a person. In addition, A(H5) serologic tests conducted in 2 states did not identify evidence of asymptomatic infection with influenza A(H5) 2.3.4.4b virus. Although some persons may have worn full PPE without breach, many probably had a PPE breach. Although the full extent of exposure among those monitored is unknown, our results are consistent with a low risk for avian-to-human transmission among persons exposed to wild and domesticated birds infected with influenza A(H5N1) clade 2.3.4.4b viruses detected in the United States.

Our findings are consistent with other reports. In previous years, US avian outbreaks with HPAI A(H5) viruses detected 0 human cases of infection with those viruses (*12*,*13*). In addition, no cases of human infection with H5N1 viruses were detected in Europe during 2016─2021, despite many avian H5N1 outbreaks (*14*,*15*).

At the time of this investigation, 7 persons exposed to the current H5N1 virus clade had H5N1 virus detected by rRT-PCR. Some of those cases were asymptomatic or mild and could represent contamination of the nasal mucosa instead of infection. Serologic testing of exposed persons in 2 states failed to find A(H5) in nasal mucosa or evidence of asymptomatic infection by antibody detection; however, the number of participants with serologic specimens was small, and a larger sample size is needed to confirm these findings.

One limitation of our study is that the number of persons exposed to H5N1-infected birds was underestimated because of underreporting and noncompliance with monitoring; however, jurisdictions requested employee lists and inquired about additional contacts to expand capture of those exposed. Detailed exposure information was not collected from all exposed persons, so we could not report on the influence of exposure duration or PPE use on infection risk.

Although we found that the risk for A(H5) virus transmission to the public appears to be low, close monitoring of these viruses and persons exposed to them is imperative. The virus is continuing to reassort with other North American avian influenza viruses, increased A(H5) cases are occurring in mammals, and the risk profile could change at any moment. Influenza A(H5N1) viruses remain a potential pandemic threat, and limiting the incidence of human zoonotic infections and human-to-human transmission is critical.

## References

[1] Centers for Disease Control and Prevention. Avian influenza in birds. 2022 [cited 2022 Aug 15]. https://www.cdc.gov/flu/avianflu/avian-in-birds.htm

[2] Yamaji R, Saad MD, Davis CT, Swayne DE, Wang D, Wong FYK, et al. Pandemic potential of highly pathogenic avian influenza clade 2.3.4.4 A(H5) viruses. Rev Med Virol. 2020;30:e2099. 10.1002/rmv.2099 32135031 PMC9285678

[3] Bevins SN, Shriner SA, Cumbee JC Jr, Dilione KE, Douglass KE, Ellis JW, et al. Intercontinental movement of highly pathogenic avian influenza A(H5N1) clade 2.3.4.4 virus to the United States, 2021. Emerg Infect Dis. 2022;28:1006–11. 10.3201/eid2805.220318 35302933 PMC9045435

[4] US Department of Agriculture Animal and Plant Health Inspection Service. USDA confirms highly pathogenic avian influenza in wild bird in South Carolina. 2022 [cited 2022 Mar 22]. https://www.aphis.usda.gov/aphis/newsroom/stakeholder-info/sa_by_date/sa-2022/hpai-sc

[5] Pan American Health Organization. Informative note: human infection caused by avian influenza A(H5) virus in Chile—31 March 2023 [cited 2023 Apr 7]. https://www.paho.org/en/documents/informative-note-human-infection-caused-avian-influenza-ah5-virus-chile-31-march-2023

[6] Centers for Disease Control and Prevention. Technical report: highly pathogenic avian influenza A(H5N1) viruses. 2023 [cited 2023 Apr 4]. https://www.cdc.gov/flu/avianflu/spotlights/2022-2023/h5n1-technical-report.htm

[7] US Department of Agriculture Animal and Plant Health Inspection Service. 2022–2023 detections of highly pathogenic avian influenza. 2023 [cited 2023 Apr 7]. https://www.aphis.usda.gov/aphis/ourfocus/animalhealth/animal-disease-information/avian/avian-influenza/2022-hpai

[8] Centers for Disease Control and Prevention. Information on bird flu. 2022 [cited 2022 Aug 15]. https://www.cdc.gov/flu/avianflu

[9] Centers for Disease Control and Prevention. Interim guidance on testing and specimen collection for patients with suspected infection with novel influenza A viruses with the potential to cause severe disease in humans. 2022 [cited 2023 Apr 17]. https://www.cdc.gov/flu/avianflu/severe-potential.htm

[10] Levine MZ, Holiday C, Liu F, Jefferson S, Gillis E, Bellamy AR, et al. Cross-reactive antibody responses to novel H5Nx influenza viruses following homologous and heterologous prime-boost vaccination with a prepandemic stockpiled A(H5N1) vaccine in humans. J Infect Dis. 2017;216(suppl_4):S555–9. 10.1093/infdis/jix001 28934456 PMC5853660

[11] Centers for Disease Control and Prevention. Current U.S. bird flu situation in humans. 2022 [cited 2022 Oct 12]. https://www.cdc.gov/flu/avianflu/inhumans.htm

[12] Olsen SJ, Rooney JA, Blanton L, Rolfes MA, Nelson DI, Gomez TM, et al. Estimating risk to responders exposed to avian influenza A H5 and H7 viruses in poultry, United States, 2014–2017. Emerg Infect Dis. 2019;25:1011–4. 10.3201/eid2505.181253 30741630 PMC6478193

[13] Arriola CS, Nelson DI, Deliberto TJ, Blanton L, Kniss K, Levine MZ, et al.; H5 Investigation Group. H5 Investigation Group. Infection risk for persons exposed to highly pathogenic avian influenza A H5 virus-infected birds, United States, December 2014–March 2015. Emerg Infect Dis. 2015;21:2135–40. 10.3201/eid2112.150904 26583382 PMC4672413

[14] Adlhoch C, Baldinelli F, Fusaro A, Terregino C. Avian influenza, a new threat to public health in Europe? Clin Microbiol Infect. 2022;28:149–51. 10.1016/j.cmi.2021.11.005 34763057

[15] Adlhoch C, Miteva A, Zdravkova A, Miškić T, Kneževic D, Perdikaris S, et al. Estimation of the number of exposed people during highly pathogenic avian influenza virus outbreaks in EU/EEA countries, October 2016-September 2018. Zoonoses Public Health. 2019;66:874–8. 10.1111/zph.12629 31493311 PMC6852165

